# Assessment of Knowledge and Attitude of Tuberculosis Patients in Direct Observation Therapy Program towards Multidrug-Resistant Tuberculosis in Addis Ababa, Ethiopia: A Cross-Sectional Study

**DOI:** 10.1155/2020/6475286

**Published:** 2020-06-06

**Authors:** Firew Tadesse Kusheno, Teklehaimanot Mezgebe Nguse, Gebremedhin Beedemariam Gebretekle

**Affiliations:** ^1^ICAP-Ethiopia, Addis Ababa, Ethiopia; ^2^Departments of Radiography, School of Medicine, College of Health Sciences, Addis Ababa University, Ethiopia; ^3^Department of Pharmaceutics and Social Pharmacy, School of Pharmacy, College of Health Sciences, Addis Ababa University, Ethiopia

## Abstract

**Background:**

Multidrug-resistant tuberculosis (MDR-TB) is becoming a major challenge of tuberculosis (TB) control program globally but more serious in developing countries like Ethiopia. In 2013, a survey result showed that in Ethiopia, tuberculosis patients from new cases and retreatment cases had resistance to at least isoniazid and rifampicin with a significant increase over time. Inadequate knowledge and wrong perception about MDR-TB by patients were detrimental to TB control programs. The study aimed at assessing the knowledge and attitude of TB patients of direct observation therapy program towards multidrug-resistant tuberculosis in health centres of Addis Ababa, Ethiopia.

**Methods:**

A cross-sectional study was conducted in 10 health centres of Addis Ababa which were selected by simple random sampling technique. A total of 422 TB patients were included in the study, and participants from each health centres were taken proportional to the number of clients in each health centres. Data was entered and analyzed using SPSS version 20. Association between outcome and independent variables was explored using logistic regression.

**Results:**

The level of knowledge of TB patients about MDR-TB was poor and only 55.0% of TB patients attained good overall knowledge. A significant association was found between good knowledge and attending tertiary level of education (AOR = 4.3, 95%CI = 1.9, 9.8), gender (AOR = 1.62, 95%CI = 1.1, 2.4), income of respondents' family (OR = 0.4, 95%CI = 0.2, 0.9), and sleeping practice (AOR = 8.0, 95%CI = 4.0, 15.7). Nearly three-fourths (73.5%) of TB patients had a favourable attitude towards MDR-TB. Occupational status (AOR = 4.4, 95%CI = 2.5, 7.6) and sleeping practices (AOR = 2.4, 95%CI = 1.2, 5.0) were significantly associated with the attitude of the TB patients.

**Conclusions:**

Knowledge of TB patients toward MDR-TB was poor. Although a large proportion of patients had a favourable attitude, it still needs to be improved. Hence, efforts should be made to implementing health education to improve awareness of TB patients about MDR-TB.

## 1. Introduction

Tuberculosis (TB) is a leading cause of morbidity and mortality of worldwide, accounting for about 9.6 million new cases and 1.5 million deaths annually [1]. In 2015, there were an estimated 10.4 million new TB cases and 1.4 million TB deaths worldwide. The higher burden of TB disease is observed among men, with ratios ranging from 1.5 (in Ethiopia) compared to 6.0 (in Rwanda) for smear-positive TB patients, and also from 1.2 (in Ethiopia) compared to 4.5 (in Vietnam) for bacteriologically confirmed TB patients [2]. The proportion was highest in the WHO African Region countries and exceeded 50% in parts of southern Africa [2]. Africa accounted for about 26% of the incident TB cases, with the highest rates of incident cases and deaths [2]. Ethiopia is one of the 30 High TB Burden Countries in the world with estimated TB incident cases of 192 per 100,000 in 2015 (WHO, 2016). Furthermore, the issue of poverty as one of the major risk factors for developing TB places Ethiopia as a high-risk environment [3].

The disease burden caused by TB is falling globally, in all WHO regions, and in most countries, but not fast enough to reach the first (2020) milestones of the End TB Strategy [4]. Nonetheless, these efforts have been jeopardized by multidrug-resistant TB (MDR-TB), and MDR-TB is caused by an organism that is resistant to at least isoniazid and rifampin which are the two most potent TB drugs [4]. In 2015, there were an estimated 480,000 new cases of multidrug-resistant TB (MDR-TB) and an additional 100,000 people with rifampicin-resistant TB (RR-TB) who were also newly eligible for MDR-TB treatment (WHO, 2016) [2]. According to the WHO report, Ethiopia also had an estimated 1700 and 550 MDR-TB cases among notified new and retreatment pulmonary TB cases in 2011, respectively [5]. A 2013 survey result showed that 2.3% of new cases and 17.8% of retreatment cases were resistant to at least rifampicin and isoniazid with a significant increase in Ethiopia [6]. As a result, drug-resistant TB has become a major challenge of the TB control program in Ethiopia [7].

Inadequate anti-TB treatment is an important factor that can contribute to the development of drug-resistant TB strains [8]. The factors causing inadequate anti-TB treatment can be grouped into health system factors (inadequate training and drug stock-outs), drug factors (high pill burden and long duration of treatment), and patient factors (poor adherence, mal-absorption, and adverse drug effects) [9]. Studies have identified that there are misconceptions and limitations of knowledge about MDR-TB among TB patients [10]. Also in this study, 150(35.5%) of TB patients agree that MDR-TB is treated by praying or holy water, 195 (46.2%) agreed that people get MDR-TB through drinking of alcohol, and 102 (24%) of TB patients agreed that taking traditional medicine can cure of MDR-TB. In Mongolia, only 31.8% of the respondents knew that TB is not heritable, and 22.7% were aware of complications and high chances of spreading the infection to family members in case of not taking treatment or discontinue the treatment [11].

The lack of knowledge about the cause, mode of transmission, and symptoms, as well as appropriate treatment of TB within communities also contribute to poor adherence to TB treatment and/or long delay in diagnosis [12]. This in return will contribute to the increasing emergence of MDR-TB and finally leaving patients with minimal choices [13]. To the best of our knowledge, no study has been conducted in Ethiopia to assess the level of knowledge and attitude of TB patients about MDR-TB. Hence, the purpose of this study was to assess the knowledge and attitude of TB patients of direct observation therapy program towards MDR-TB in health centres of Addis Ababa, Ethiopia. The findings could be used by policymakers to designing appropriate prevention and control strategies.

## 2. Methods

### 2.1. Study Area

The study was conducted in public health centres in Addis Ababa, Ethiopia. Addis Ababa is the capital city of Ethiopia and the political hub of Africa. Administratively, the city is divided into 10 subcities and 116 Woredas. It has a total area of 54,000 hectares, and the total population is 3,122,000 of which 1,634,403 (52.6%) of them are females (Central Statistical Agency Ethiopia), Population Projection of Ethiopia for all regions at Woreda level from 2014–2017 Addis Ababa, Ethiopia, 2013 [14]. The city administration has a total of 93 health centres and 11 public hospitals [7].

### 2.2. Study Design

A facility-based cross-sectional study was conducted in public health centres of Addis Ababa between April 20 and May 30, 2016. TB Patients under the direct observation therapy (DOT) program in all health facilities of Addis Ababa were the source of the study. All TB patients, who attended the DOT program in public health centres between April 20 and May 30, 2016, were sources of the data for the study. However, all TB patients who attended the DOT program in hospitals and private health institutions were excluded from the study.

The sample size was determined by using a single proportion formula [15]. Due to absence of research related to knowledge, nonchalant attitude toward MDR-TB among the DOT program in Ethiopia, and difficulties in getting maximum sample size, it was assumed that about 50% of TB patients had good knowledge and favourable attitude toward MDR-TB. The margin of error was assumed to be 5% with a 95% confidence interval. Thus, considering a 10% none and inappropriate response rate, a total of 422 TB patients were included in this study.

From the ten subcities in Addis Ababa, five subcities were selected by simple random sampling method. Then, a total of ten health centres, two from each subcity, were selected using a random sampling method. Finally, the number of TB patients to be recruited from each health centres were decided based on the size of patients attending the DOT program, and individual participants from each health facility were selected using a random sampling method.

### 2.3. Data Collection and Management

Data were collected using semistructured questionnaires adopted from different scholars and modified based on the current research [6, 7, 9, 10]. The questionnaire had three parts. The first part was designed to elicit the sociodemographic characteristics of each study participant, while the second part was related to knowledge and twelve questions were provided. In the third part, attitudes of study participants were assessed by 12 questions, which focused on symptoms, transmission, prevention, and treatment of MDR-TB. The dependent variables of the study were knowledge and attitude of TB patients towards MDR-TB. Gender, age, educational status, marital status, occupational status, overcrowding (family size), use of ventilation system, and average monthly income of respondents were identified as an independent variable of the study.

The data were collected by face-to-face interview and ten health professionals; 5 health officers, 3 Bachelor of Science, and 2 diploma nurses who were not working in the selected health facilities were recruited as data collectors. Before the actual data collection, one-day training focused on the aim of the study, and a detailed review of the tool was given to all data collectors. The questionnaires were preadministered in 22 (5%) of TB patients in one of the health centres which was not included in the actual study and modification was made accordingly. Furthermore, adequate supervision and follow up was done by the supervisors to maximize the quality of the data collected.

### 2.4. Data Entry and Analysis

Data were entered and cleaned using EPI-info version 3.5.1 and exported to SPSS version 20.0 for analysis. Simple descriptive statistics including mean, percentage, and standard deviations were computed to summarize categorical variables. Possible associations between the variable of interest were explored using logistic regression as significant at *p* < 0.05.

Giving correct answers earned a score of 1 but if the response is wrong, then it attracts a score of 0. Knowledge and attitude for individuals were calculated and summarized to give total score. Sum up to give their total score. In this study, knowledge was measured based on the ability of patients to correctly identify and respond to cause, mode of transmission and factors related to transmission, signs and symptoms, and possible ways of prevention of TB and MDR-TB treatment. Then, knowledge aspects were categorized into good and poor score if it is equal or above the mean and below the mean, respectively. The attitude was measured by feelings towards the cause, treatment, and about the follow-up and felling when others knew that you have TB. Each had one point if correctly answered. The favorability and unfavorability classification was based on each mean value. Participants who scored equal or above the mean were considered favourable attitude, if it is below considered as unfavourable attitude.

### 2.5. Ethical Consideration

The study was approved by the Addis Ababa City Administration Health Bureau (ref. no. A/A/H/6381/227) and Africa Medical College ethics review board. Besides, permission was sought from the subcities and respective health centres. Finally, informed verbal consent was obtained from all participants after explaining the purpose of the study and informing that their response will be kept confidential. Questionnaires were coded, and collected data was locked in a lockable cabinet to maintain the confidentiality of the information obtained.

## 3. Results

### 3.1. Sociodemographic Profile of the Studied Participants

A total of 422 TB patients were interviewed in this study. Among them, 217 were male which contributed 51.4% for the total analysis, and 50.2% of them were ever married. More than two-thirds (67.3%) of patients were less than or equal to 34 years, and the mean age of respondents was 32.12 ± 11.0 years. The interviewed people in terms of age ranged from 13 to 70 years, as shown in Table 1.

Concerning their educational status, the majority of 147 (34.8%) of the TB patients attended primary level followed by the tertiary level of education which accounted for 140 (33.2%). Regarding their average monthly family income, 175 (41.5%) of them had only ≤585 ETB. The study also revealed that 293(69.4%) of the TB patients were employed. Concerning their living condition, 76 (18%) of them were living with five or more of his/her family members or friends. More than one-fourth (27.7%) of TB patients were sharing one bed with a member of the family or friend in one room (Table 1).

From the 422 TB patients, 134 (33.0%) never heard about MDR-TB at all, while 178 (62.7%) heard about MDR-TB from health workers. Regarding the definition of MDR-TB, 68 (16.1%) of TB patients said they know the meaning of MDR-TB as TB disease caused by a strain of mycobacterium tuberculosis that is resistant to at least two (Isoniazid and Rifampicin) anti-TB drugs. On the other hand, 70 (16.6%) of TB patients did not know the meaning of MDR-TB. Regarding the high-risk group of developing MDR-TB, 53 (12.6%) of TB patients did not know who was at high risk to develop MDR-TB. Respondents were also asked on the possible consequences of defaulting anti-TB treatment, and 146 (34.1%) of TB patients correctly mentioned that TB patients may develop MDR-TB, and 56 (13.3%) of TB patients believed that repeated interruption of treatment leads to more drug resistance and treatment failure. One hundred and thirteen (26.8%) respondents reported that poor people are at a higher risk of developing MDR-TB compared to wealthy people (Table 2).

Regarding the causes of MDR-TB, 212 (50.2%) of TB patients said that the causes of MDR-TB are bacteria or germs that cannot be seen with the naked eyes, while 151 (35.8%) respondents said that the causes of MDR-TB were cold weathers, God acrimony/penalty and virus, respectively. Overall, almost half (49.8%) of TB patients did not know the exact causes of MDR-TB. Concerning transmission of MDR-TB, 361 (85.5%) of the TB patients mentioned that through inhalation of infected droplet nuclei during coughing, sneezing, and talking was the way of transmission, 38 (9%) said that sexual contact, while 42 (9.9%) said it can be transmitted through blood contact. Regarding the duration of treatment of MDR-TB, 217 (51.3%) TB patients knew the duration of treatment of MDR-TBs which is between 18 to 24 months, and 69 (16.4%) of TB patients did not know the duration of treatment of MDR-TB. Regarding the possible ways to control the transmission of MDR-TB, 101 (24.0%) of TB patients reported that it was not possible to control the transmission of MDR-TB while the rest of the respondents knew one or more controlling mechanisms from the given choices (Table 2).

TB patients' overall knowledge of MDR-TB was evaluated by summarizing the twelve questions, and the study showed that the mean knowledge scored was 6.0 ± 0.5. The study found that 190 (45.0%) of patients scored below the mean, and they had poor knowledge about MDR-TB. Multivariate analysis showed a significant association of overall knowledge with an educational status where those with tertiary level educational status had more likely of good knowledge (AOR = 4.3, 95%CI = 1.9, 9.8) compared to the illiterate patients. Male TB patients were 1.6 times more likely to have good knowledge than females. Knowledge of TB patients was also significantly associated with the sleeping practice where TB patients sleeping alone in a separate room (AOR = 8.0, 95%CI = 4.0, 15.7) and sleeping alone but not separate room (AOR = 5.8, 95%CI = 2.9, 11.2) were more likely to have good knowledge compared to those who were sleeping in one bed with his/her family or friends. Respondents whose family average monthly income less than 585 ETB had poor overall knowledge (AOR = 0.4, 95%CI = 0.2, 0.9) compared with respondents whose family average monthly income greater than or equal to 3146 ETB (Table 3). On the other hand, multivariate analysis showed that age, marital status, family size, and occupational status of respondents were not significantly associated with knowledge of patients about MDR-TB (Table 3).

### 3.2. TB Patients Overall Attitude towards MDR-TB and Its Associated Factors

All respondents were asked about their attitudes towards MDR-TB, as presented in Table 4. More than one-third (37.9%) of TB patients agreed that they had fear of discrimination and 150 (35.5%) of the respondents agreed that MDR-TB can be treated by praying or by holy water. The study also showed that 195 (46.2%) of TB patients expressed their agreement that someone can acquire MDR-TB through drinking alcohol or smoking. Regarding the treatment of MDR-TB, 102 (24%) of the respondents agreed that taking traditional medicines can cure MDR-TB and 296 (70.1%). The patients revealed that taking TB treatment under direct observation of health professionals is an important way to prevent the development of MDR-TB. Also, 102 (24%) respondents agreed that someone could stop taking TB treatment when he/she feels better while 141 (33.4%) agreed that the treatment of TB should be stopped when they encountered side effects of the drug. From all studied participants, 192 (45.5%) of TB patients agreed that the MDR-TB is caused by human activities and 321 (76.1%) of the TB patients agreed that increasing the prevalence of MDR-TB in Ethiopia has high impact in social, political, and economic development. On the other hand, 273 (64.7) of TB patients agreed that MDR-TB is a highly infectious and contagious disease (Table 4).

The study showed that the mean attitude scored of TB patients was 6 ± 0.4. One hundred twelve (26.5%) of TB patients scored below the mean and had unfavourable attitudes about MDR-TB. Multivariate analysis results also revealed that there was a significant relationship between the attitude of MDR-TB and occupational status of respondents where those who were unemployed had more unfavourable attitudes towards MDR-TB (AOR = 4.4, 95%CI = 2.5, 7.6) compared to employed ones. Respondents who sleep alone in a separate room had a favourable attitude (AOR = 2.4, 95%CI = 1.2, 5.0) as compared to TB patients who sleep in one bed with his/her family or friends. The study also showed a significant relationship between the attitude of respondents about MDR-TB and monthly family income. TB patients with an average monthly family income of less than or equal to 585 Ethiopian Birr (AOR = 2.0, 95%CI = 1.03, 3.9) and 586-1650 Ethiopian Birr (AOR = 2.9, 95%CI : 1.2, 6.6) had more favourable attitudes than those who had a monthly family income of ≥3146 Ethiopian Birr. On the other hand, the attitude of respondents about MDR-TB was not significantly associated with gender, marital status, and educational status of TB patients (Table 5).

## 4. Discussion

From 422 TB patients, only 284 (67.9%) TB patients ever heard about MDR-TB, and of these, only 178 (62.7%) of them heard it from healthcare professionals which are less than a study conducted in Southwest Ethiopia in which 83% of TB patients heard about MDR-TB. This could be due to the insufficiency in the provision of health education about MDR-TB that should be given to all health centres, and TB patients might also assume themselves as knowledgeable [16]. Additionally, healthcare professionals' poor knowledge about MDR-TB might contribute to lower patient knowledge about the disease as they may convey inadequate and incomplete information about the disease and treatment regimen [10, 17].

In this study, the overall knowledge of TB patients was not satisfactory as only 55% of TB patients had good knowledge about MDR-TB. This was not unexpected because many studies in Africa and other parts of the world have documented that TB patients lack basic knowledge of aetiology, transmission, prevention, and duration of treatment of MDR-TB [17–19], but it is better than the study conducted in Nigeria in which only 18.4% of patients had good knowledge of MDR-TB [10]. The poor knowledge of TB patients is inimical to the control of the disease in any population. It is also a common practice for patients to seek information from other sources like neighbours, traditional healers, and churches. This at times might worsen their condition by creating the wrong perception about the disease and unsubstantiated health-seeking behaviours. In the case of TB, it has been shown in many studies that poor knowledge and wrong perceptions were responsible for the delay in seeking health care in a health facility, treatment default, and stigmatization of TB patients [4, 18, 20, 21]. All these were contributing factors for the rising prevalence of MDR-TB, and this poses a major challenge to many National TB Control Programs. Thus, the Federal Ministry of Health and other stakeholders should make a concerted effort to strengthen the provision of education during the DOT period and organize awareness creation campaigns to improve the knowledge and attitude of TB patients.

The finding of this study revealed that the level of knowledge about MDR-TB was positively associated with the educational status of TB patients. The odd of good knowledge in TB patients attended tertiary level of education was 4.3 times higher than the odds of TB patients who did not attend any level of education or illiterates. This was attributed to relatively better awareness about MDR-TB and better access to health information in those attended tertiary level of education (28.4%) in Addis Ababa, Ethiopia, compared with a study conducted in Delta state, Nigeria (19.7%), and the study conducted in Ulaanbaatar (53.9% of patients did not know about TB) [10, 11].

In this study, knowledge had a significant association with the sleeping practice of studied participants. The present study demonstrated that the likelihood of having good knowledge was 1.3 times higher in those patients who sleep alone in a separate room than respondents who shared a bedroom with his/her family or friends. On the contrary, a study conducted in Yangon Myanmar found that 96.3% of patients did not sleep with family members under the same net [22]. The current findings might be due to a lack of awareness and knowledge about the mode of transmission and ways of prevention of MDR-TB. This fact has been supported by the present finding where 65 (15%) of the study participants mentioned that TB cannot be transmitted by inhalation of an infected droplet from person to person and 80 (18.9%) of them also wrongly mentioned that TB can be transmitted via blood and sexual contacts.

From 422 TB patients, more than one-fourth (26.5%) have unfavourable attitudes towards MDR-TB whereas 32.5% were confirmed in the study conducted in Delta state, Nigeria [9]. The attitude developed among the people could be the reason for defaulting anti-TB drug, stop MDR-TB treatment due to minor side effect, stop taking TB treatment drug when he/she feels better, and take traditional medicines to cure of MDR-TB.

In this study, 205 (48.5%) of TB patients disagreed by the cause of MDR-TB is man-made and 150 (35.5%) respondents believed that MDR-TB is treated by praying or by holy water. This indicated that TB patients have misconceptions, creating wrong perception, and negative health-seeking behaviours about MDR-TB [10].

In the current study, 321 (76.1%) respondents agreed that increasing the prevalence of MDR-TB in Ethiopia has a high impact in social, political, and economic development, whereas 76 (18.0%) respondents did not agree. The 296 (70.1%) respondents agreed on taking TB treatment by direct observation of health personnel is an important way of developing MDR-TB which has a less favourable attitude about MDR-TB compared with the study conducted on Amhara region in Ethiopia showed that 95.7% [23].

From all TB patients who have the favourable attitude, 177(57.1%) TB patients have not heard about the disease from health care workers which revealed inadequate or incomplete information passed on to the TB patients by HCWs would create wrong perceptions in them or strengthen the patients' perceptions which were similar with the study conducted by Delta State, Nigeria [10], to solve this preparation of IEC and BCC materials and expanding IEC activities using the TB patients concerning MDR-TB. So those TB patients use as cadres to advocate about MDR-TB.

## 5. Limitations and Strengths

The limitation of this study; the research was conducted only on patients who were diagnosed and taking anti-TB treatment on the DOT program and not measuring the practice of TB patients. The strength of the study; the project was focused on different subcities and health centres with trying to address patients' related factors influencing MDR-TB prevention and control strategies.

## 6. Conclusion

This study identified that the knowledge of TB patients toward MDR-TB was poor (45%), one-third of TB patients (33.0%) never heard about MDR-TB, and two-third (62.7%) of TB patients heard from health workers and the number of studied patients also showed unfavourable attitude (26.5%) towards MDR-TB in Addis Ababa, Ethiopia.

The different factors which affect the knowledge and attitude of TB patients about MDR-TB were occupational status, average monthly income, sleeping practices, educational status, and others. Subsequently, it is essential to improve the living condition of TB patients, and efforts should be made to implement health education to improve awareness of TB patients about MDR-TB.

## Figures and Tables

**Table 1:** Sociodemographic and socioeconomic profiles of TB patients in Addis Ababa, Ethiopia, 2016 (*N* = 422).

Characteristics	*n* (%)
Age	
≤34 years	280 (67.3)
35-54 years	115 (27.6)
>54 years	21 (5.0)
Educational status	
Illiterate	40 (9.5)
Primary level (1 to 8 grade)	147 (34.8)
Secondary level (9 to12 grade)	95 (22.5)
Tertiary level	140 (33.2)
Marital status	
Ever married	214 (50.2)
Never married	207 (49.8)
Occupational status	
Unemployed	129 (30.6)
Employed	293 (69.4)
Average monthly income of respondents' family (ETB)	
<585	175 (41.7)
586-1650	58 (13.8)
1651-3145	82 (19.5)
≥3146	105 (25.0)
Number of family or friends living with TB patients	
No one with me	102 (24.2)
2-4 family/friend's size	244 (57.8)
≥5 family/friend's size	76 (18.0)
Sleeping practice of TB patients	
Alone and separate room	189 (44.8)
Alone but not separate room	116 (27.5)
Use one bed with others in one room	117 (27.7)

**Table 2:** Frequency and percent of knowledge measuring variables of TB patients, Addis Ababa, Ethiopia, 2016 (*N* = 422).

Characteristics	Frequency (%)
Where have you heard about MDR-TB?^∗^	
Heard from health workers	178 (42.2)
Heard from mass media (television, radio, and taps)	94 (22.3)
Heard from printed or electronic media	29 (6.9)
Heard from friends, family, and school	49 (11.6)
What are the possible consequences of defaulting on TB treatment?^∗^	
The TB is not cured and it will come back again	301 (71.3)
TB patient may die	171 (40.5)
Patient may develop multidrug-resistant TB (MDR-TB)	144 (34.1)
Longer duration of absence from patient's job (source of income)	61 (14.5)
Not know	9 (2.1)
What is the meaning of multidrug resistance tuberculosis?^∗^	
Tuberculosis disease caused by a strain of TB that is resistant to at least two anti-TB drugs.	68 (16.1)
Tuberculosis disease that is resistant to one anti-TB drug	77 (18.2)
Form of TB that requires treatment with expensive drugs	60 (14.2)
Form of TB that requires treatment which gives more side effects	40 (9.5)
Resistance due to default of anti TB or not complete anti-TB drug.	193 (45.7)
Not know meaning of MDR-TB.	70 (16.6)
What is the cause of multidrug resistance tuberculosis?	
Bacteria	212 (50.2)
Virus and not know cause	66 (15.7)
From GOD acrimony/penalty	27 (6.4)
Cold weather	151 (35.3)
What are the common transmission ways of MDR-TB?	
Person to person (inhalation of infected droplet nuclei during coughing, sneezing	357 (85.5)
By sexual contact	38 (9.0)
By blood contact and (others)	42 (9.9)
What are the signs and symptom of tuberculosis?^∗^	
Long-lasting cough (two weeks and greater)	371 (87.9)
Persistent fever	193 (45.7)
Loss of weight	192 (45.5)
Hemoptysis	208 (49.3)
Night sweating	183 (43.4)
Chest pain	90 (21.3)
Loss of appetite	120 (28.4)
Others like swelling, weakness, rheumatism	18 (4.3)
What is the duration of treatment of MDR-TB?	
Duration of treatment of MDR-TB (6 months and 8 months)	136 (32.3)
Duration of treatment of MDR-TB (18, 20, and 24 months)	217 (51.3)
Not know duration of treatment of MDR-TB	69 (16.4)
Why was it important to complete treatment of tuberculosis?^∗^	
TB patients to be cured	386 (91.5)
Repeated interruption of treatment leads to drug resistance and treatment failure	56 (13.3)
It control/prevent from transmission of TB	136 (32.3)
If the treatment is not completed, the TB has a high chance of coming back and untreated TB can result in death	113 (26.8)
Who is high risk to develop MDR-TB?^∗^	
Retreatment regimen failure TB patient	194 (46.0)
Close contact history with a known MDR-TB patient	142 (33.6)
New treatment regimen failure TB patient	137 (32.5)
Retreatment TB patients [e.g., return after default, relapse)	97 (23.0)
Under five children are high risk	102 (24.2)
People living with HIV/ADIS	89 (21.1)
Not know who was high risk to develop MDR-TB	53 (12.6)
Others like health workers, stopped TB treatment drugs	7 (1.7)
Was it possible to control the transmission of MDR TB?	
Yes	321 (76.1)
No	101 (24)
How can it is possible to prevent MDR-TB?^∗^	
Completing all TB cases treatment properly without defaulting	266 (63)
Cover mouth and nose when cough, sneezing, laughing and talking	139 (33)
Keep the windows and doors opened	144 (34.1)
By using the appropriate treatment regimen/drug for all TB cases.	87 (20.6)
Which group of people do you think affected by MDR-TB mostly?	
Rich	6 (1.4)
Poor	113 (26.8)
Anybody	303 (71.8)

^∗^ indicates percentage sum was more than 100% because of multiple responses and think that there are missed value in variables.

**Table 3:** TB patients' overall knowledge and associated factors on MDR-TB in Addis Ababa, Ethiopia, 2016 (*N* = 422).

Characteristics	Overall knowledge (*n* (%))		Odds ratio	
	Poor	Good	Crude (COR)	Adjusted (AOR)
Sex				
Male	84 (19.9)	133 (31.5)	1.7 (1.2, 2.5)	1.62 (1.1, 2.4)^∗^
Female	106 (25.1)	99 (23.5)	1.0	1.0
Age (years)				
≤34	124 (29.4)	156 (36.9)	1.0	1.0
35-54	55 (13.0)	60 (14.2)	0.87 (0.56, 1.34)	0.81 (0.5, 1.3)
≥55	8 (1.9)	13 (3.1)	1.3 (0.52, 3.22)	1.2 (0.5, 2.9)
Marital status				
Never married	92 (18.7)	115 (31.1)	1.1 (0.7, 1.55)	0.9 (0.6, 1.5)
Ever married	98 (26.3)	116 (23.9)	1.0	1.0
Educational status				
None (illiterate)	24 (5.7)	16 (3.8)	1.0	1.0
Primary level	79 (18.7)	68 (16.1)	1.3 (0.634, 2.64)	1.4 (0.6, 3.0)
Secondary level	43 (10.2)	52 (12.3)	1.8 (0.86, 3.8)	2.2 (1.0, 5.2)
Tertiary level	44 (10.4)	96 (22.8)	3.3 (1.6, 6.8)	4.3 (1.9, 9.8)^∗∗^
Occupational status				
Unemployed	65	64	1.0	1.0
Employed	125	168	1.4 (0.8, 2.1)	0.9 (0.6, 1.5)
Average monthly income of respondent's family or friends (ETB)				
<585	85 (20.1)	81 (19.2)	0.6 (0.4, 1.1)	0.5 (0.2, 1.3)
586-1650	30 (7.1)	36 (8.5)	0.4 (0.2, 0.7)	0.4 (0.2, 0.9)^∗^
1651-3145	39 (9.2)	44 (10.4)	0.4 (0.23, 0.7)	0.6 (0.3, 1.3)
≥3146	35 (8.3)	70 (16.6)	1.0	1.0
Sleeping practice in your home				
Alone and separate room	69 (16.4)	120 (28.4)	3.5 (2.14, 5.65)	8.0 (4.0, 15.7)^∗∗^
Alone but not separate room	43 (10.2)	73 (17.3)	3.4 (2.0, 5.82)	5.8 (2.9, 11.2)^∗∗^
In one bed with other members of family or friends	78 (18.5)	39 (9.3)	1.0	1.0
No. of family/friends live with TB patients				
No one with me	39 (9.2)	63 (14.9)	1.7 (1.01, 2.8)	1.9 (0.7, 4.5)
2-3 members	69 (16.4)	90 (21.3)	1.2 (0.7, 1.9)	1.2 (0.6, 2.2)
≥4 members	82 (19.4)	79 (18.7)	1.0	1.0

^∗^ indicates statistical significance association *b*/*n* independent and dependent variable. ^∗∗^ indicates statistical strongly significance association *b*/*n* independent and dependent variables. ^∗^ indicates percentage sum was more than 100% because of multiple responses and think that there are missed value in variables.

**Table 4:** Frequency and percent of attitude measuring variables of TB patients about MDR-TB, Addis Ababa, Ethiopia, 2016 (*N* = 422).

Characteristics	Agree	Neutral	Disagree
There was fear of discrimination due to TB patient	160 (37.9%)	19 (4.5%)	243 (57.6%)
The cause of MDR-TB is man-made	192 (45.5%)	26 (6.2%)	204 (48.3)
MDR-TB can be treated by praying or by holy water	150 (35.5%)	38 (9%)	234 (55.5%)
People can acquire MDR-TB through drinking alcohol and/or smoking cigarette	195 (46.2%)	38 (9%)	189 (44.8%)
Covering mouth and nose by the mask is one way of protecting the transmission of MDR-TB to other people	337 (79.9%)	11 (2.6%)	74 (17.5%)
Taking traditional medicines can cure MDR-TB	102 (24%)	36 (8.5%)	284 (67.3%)
Increasing the prevalence of MDR-TB in Ethiopia has a high impact on social, political, and economic development	321 (76.1%)	25 (5.9%)	76 (18%)
Someone can stop taking anti-TB drugs when he/she feels better	102 (24.2%)	14 (3.3%)	306 (72.5%)
People do not respect you; if you are MDR-TB patient/client	149 (35.3%)	25 (5.9%)	248 (58.8%)
Someone can stop MDR-TB treatment when s/he encountered with the side effect of the drugs	141 (33.4%)	23 (5.5%)	258 (61.1%)
Taking TB treatment by direct observation of health personnel is an important way to prevent development MDR-TB.	296 (70.1%)	23 (5.5%)	103 (24.4)
Multidrug resistance tuberculosis is a highly infectious and contagious disease.	273 (64.7%)	13 (3.1%)	136 (32.2%)

**Table 5:** TB patients overall attitudes and associated factors on MDR-TB in Addis Ababa, Ethiopia, 2016 (*N* = 422).

Characteristics	Overall attitude (*n* (%))		Crude (COR)	Adjusted (AOR)
	Unfavourable	Favourable		
Sex				
Male	54 (12.8)	163 (38.6)	1.0	1.0
Female	58 (13.7)	147 (34.8)	1.2 (0.7, 1.8)	1.2 (0.7, 1.9)
Age (years)				
≤34	68 (16.1)	212 (50.2)	3.1 (0.7, 13.4)	2.9 (0.6, 12.8)
35-54	39(9.2)	76(18.0)	4.9(1.1, 22.0)	4.8(1.1, 22.1)∗
≥55	2 (0.5)	19 (4.5)	1.0	1.0
Marital status				
Never married	54 (12.8)	153 (36.3)	1.0	1.0
Ever married	57 (13.5)	157 (37.2)	1.1 (0.67, 1.6)	0.9 (0.5, 1.4)
Educational status				
None (illiterate)	14 (3.3)	26 (6.2)	1.3 (0.6, 2.6)	1.2 (0.5, 2.7)
Primary level	34 (8.1)	113 (26.8)	0.7 (0.4, 1.1)	0.7 (0.4, 1.2)
Secondary level	22 (5.2)	73 (17.3)	0.7 (0.4, 1.3)	0.6 (0.3, 1.2)
Tertiary level	42 (9.9)	98 (23.2)	1.0	1.0
Occupational status				
Unemployed	53 (12.6)	76 (18.0)	3.1 (1.9, 5.1)	4.4 (2.5, 7.6)^∗∗^
Employed	59 (13.9)	65 (15.5)	1.0	1.0
Average monthly income of respondents' family or friends				
≤585	50 (11.8)	125 (29.5)	1.7 (0.9, 3.1)	2.0 (1.03, 3.9)^∗^
586-1650	22 (5.2)	36 (8.5)	2.6 (1.3, 5.3)	2.9 (1.2, 6.6)^∗^
1651-3145	19 (4.5)	63 (14.9)	1.3 (0.6, 2.6)	1.5 (0.6, 3.2)
>3146	20 (4.8)	85 (20.1)	1.0	1.0
Sleeping practice in your home				
Alone and separate room	44 (10.4)	145 (34.4)	1.0	1.0
Alone but not separate room	28 (6.6)	88 (20.8)	1.1 (0.6, 1.8)	1.2 (0.7, 2.2)
In one bed with other members of family or friends	40 (9.6)	77 (18.2)	1.7 (1.03, 2.9)	2.4 (1.2, 5.0)^∗^
No. of family/friends live with TB patients				
No one with me	25 (5.9)	77 (18.3)	1.0	1.0
Family/friends size is 2-4	69 (16.4)	175 (41.5)	1.2 (0.7, 2.1)	1.3 (0.6, 3.5)
Family/friends size ≥5	18 (4.3)	58 (13.7)	0.96 (0.5, 1.92)	0.9 (0.3, 2.4)

^∗^ indicates statistical significance association *b*/*n* independent and dependent variable. ^∗∗^ indicates statistical strongly significance association *b*/*n* independent and dependent variable.

## Data Availability

Data supporting the findings can be found in the Tables.

## Abbreviations

BCC: Behavior change communication
DOT: Direct observation therapy
DR-TB: Drug resistance tuberculosis
ETB: Ethiopian birr
HCWs: Health care workers
IEC: Information education and communication
MDR-TB: Multidrug resistance tuberculosis
TB: Tuberculosis
WHO: World Health Organization.

## Additional Points

*Statement of the Author*. We hereby declare and affirm that this thesis is our work. All scholarly matter that is included in the thesis has been given recognition through citation.
